# Supplementary material for: Exploring the corrosion inhibition capability of FAP-based ionic liquids on stainless steel
Source: R Soc Open Sci. 2020 Jul 1;7(7):200580. doi: 10.1098/rsos.200580 (PMC7428245; doi:10.1098/rsos.200580)
Supplement: EDX Data;CA Data;Tafel Plot [file rsos200580supp1.docx]

***R. Soc. open sci.* article template**

***
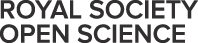
***

*R. Soc. open sci.*

doi:10.1098/not yet assigned

**Exploring the Corrosion Inhibition Capability of [FAP]- based**

**Ionic Liquids on Stainless Steel**

*Julius Kim A. Tiongson ^a^ , Kim Christopher C. Aganda ^b^,*

*Albert P. Guevara ^b^, Dwight Angelo V. Bruzon^c^, Blessie A. Basilia^b^, Giovanni A. Tapang^c^, and Imee Su Martinez^*, a, d^*

^a^ Natural Sciences Research Institute, University of the Philippines Diliman, Quezon City, Philippines, 1101

^b^ Advanced Device and Materials Testing Laboratory, Department of Science and Technology Compound, Gen. Santos Ave., Bicutan, Taguig City, Philippines, 1631

^c^ National Institute of Physics University of the Philippines Diliman, Quezon City, Philippines, 1101

^d^Institute of Chemistry, University of the Philippines Diliman, Quezon City, Philippines, 1101

**Corresponding author:* [*ismartinez@up.edu.ph*](mailto:ismartinez@up.edu.ph)

**Supporting Information**

S-I. Chemical composition of S30400 and S20200 substrates based on EDX result.

|  | S30400 | | S20200 | |
| --- | --- | --- | --- | --- |
| Elemental content (wt %) | Before acid exposure | After acid exposure | Before acid exposure | After acid exposure |
| C | 4.0 ± 0.2 | 9.2 ± 0.8 | 6.4 ± 0.2 | 19.1 ± 1.6 |
| Fe | 69.1 ± 0.1 | 66.8 ± 1.7 | 72.0 ± 0.5 | 28.1 ± 3.4 |
| Mn | -- | 10.0 ± 0.1 | 10.8 ± 0.2 | 4.4 ± 0.6 |
| Cr | 18.8 ± 0.1 | 7.9 ± 0.2 | 8.5 ± 0.1 | 2.4 ± 0.2 |
| Si | 0.5 ± 0 | 0.3 ± 0 | 0.3 ± 0.1 | 0.3 ± 0.1 |
| Ni | 7.6 ± 0.1 | 1.1 ± 0.1 | 1.3 ± 0.1 | 0.1 ± 0 |
| O | -- | 4.9 ± 1.4 | -- | 45.6 ± 5.6 |

S-II. Chemical composition of [PMIM][FAP]-coated stainless steel substrates based on EDX results. A. Ionic liquid coating before corrosion; B. Ionic liquid coating after acid exposure; C. Initially ionic liquid-coated surface after acid exposure.

|  | SS 304 | | | SS 202 | | |  |
| --- | --- | --- | --- | --- | --- | --- | --- |
| Elemental content (wt %) | A. | B. | C. | A. | B. | C. | |
| C | 36.9 ± 0 | 29.0 ± 1.4 | 5.6 ± 3.1 | 37.9 ± 0 | 29.8 ± 5.8 | 8.2 ± 1.0 | |
| F | 26.5 ± 0 | 20.9 ± 4.7 | -- | 26.6 ± 0 | 21.2 ± 1.4 | -- | |
| Fe | 26.1 ± 0 | 24.6 ± 4.1 | 67.7 ± 1.2 | 24.7 ± 0 | 33.0 ± 5.3 | 68.8 ± 2.0 | |
| Mn | 3.8 ± 0 | 2.8 ± 1.8 | 10.0 ± 0.4 | 3.8 ± 0 | 5.2 ± 0.7 | 9.8 ± 0.5 | |
| P | 3.3 ± 0 | 2.0 ± 0.4 | 0.1 ± 0 | 3.0 ± 0 | 1.5 ± 0.3 | -- | |
| Cr | 3.3 ± 0 | 3.8 ± 0.1 | 9.1 ± 1 | 3.4 ± 0 | 4.2 ± 0.5 | 8.1 ± 0.3 | |
| Si | 0.2 ± 0 | 0.2 ± 0.1 | 0.8 ± 0.4 | 0.2 ± 0 | 0.2 ± 0 | 0.4 ± 0.1 | |
| Ni | -- | -- | 1.2 ± 0.2 | 0.4 ± 0 | 0.5 ± 0.1 | -- | |
| O | -- | 14.0 ± 2.1 | 4.4 ± 0.6 | -- | 4.4 ± 1 | 4.8 ± 1.8 | |

S-III. Chemical composition of [MOBMIM][FAP]-coated stainless steel substrates based on EDX results. A. Ionic liquid coating before corrosion; B. Ionic liquid coating after acid exposure; C. Initially ionic liquid-coated surface after acid exposure.

|  | SS 304 | | | SS 202 | | |
| --- | --- | --- | --- | --- | --- | --- |
| Elemental content (wt %) | A. | B. | C. | A. | B. | C. |
| C | 44.2 ± 0.9 | 40.6 ± 5.6 | 11.6 ± 5.3 | 34.8 ± 0 | 43.6 ± 4 | 7.6 ± 0.2 |
| F | 39.5 ± 0.3 | 48.5 ± 9.9 | -- | 27.9 ± 0 | 34.9 ± 0.1 | -- |
| Fe | 4.9 ± 4.1 | 0.8 ± 0.1 | 67.1 ± 2.5 | 26.2 ± 0 | 6.6 ± 5.8 | 68.2 ± 0.4 |
| Mn | 1.5 ± 0 | -- | 9.7 ± 0.9 | 4.2 ± 0 | 2.3 ± 0 | 10.2 ± 0.1 |
| N | 6.5 ± 0 | 7.2 ± 0 | -- | -- | 8.5 ± 0 | -- |
| P | 5.8 ± 0.2 | 5.5 ± 0.3 | -- | 3.2 ± 0 | 5.3 ± 0.4 | -- |
| Cr | 1.5 ± 0 | -- | 8.0 ± 0.8 | 3.6 ± 0 | 1.1 ± 1 | 8.1 ± 0 |
| Si | -- | -- | 0.3 ± 0.1 | 0.1 ± 0 | 0.1 ± 0 | 0.3 ± 0 |
| Ni | -- | -- | 1.0 ± 0.3 | -- | -- | 1.0 ± 0.2 |
| O | 1.9 ± 0 | 2.3 ± 0 | 1.2 ± 0.1 | -- | 3.1 ± 0.6 | 3.8 ± 0.1 |

S-IV. Contact angle of water and the FAP-based ionic liquids on the stainless steel samples

| **Liquid sample** | **S30400** | |  | **S20200** | |  | |
| --- | --- | --- | --- | --- | --- | --- | --- |
|  | Initial CA (deg)^a^ | Final CA (deg)^b^ | Difference | Initial CA (deg)^a^ | Final CA (deg)^b^ | Difference (deg) | |
| Water | 78.3 ± 1.4 | 68.2 ± 1.3 | 10.0 | 73.8 ± 0.8 | 61.5 ± 0.7 | 12.2 | |
| [PMIM][FAP] | 38.8 ± 0.8 | 36.6 ± 1.3 | 2.2 | 31.7 ± 0.7 | 24.1 ± 0.4 | 7.6 | |
| [MOBMIM][FAP] | 44.9 ± 1.8 | 43.1 ± 1.4 | 1.9 | 24.6 ± 1.1 | 22.0 ± 0.3 | 2.5 |  |

^a^ Obtained at ≤ 1 minute

^b^ Obtained after 10 minutes


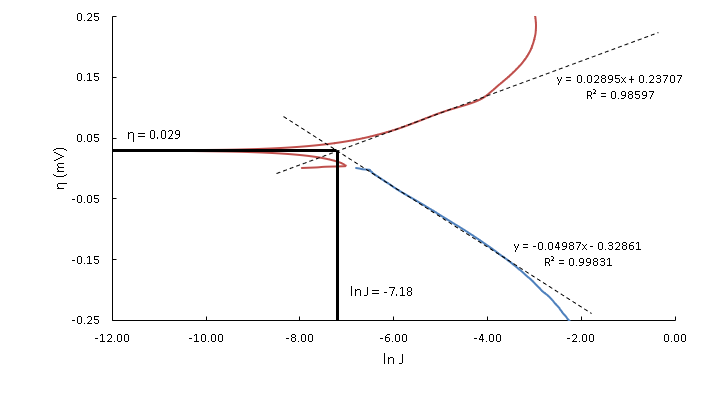


S-V. Tafel plot for the anodic and cathodic scans for 1.0 M HCl using a stainless steel working electrode.
